# Superstitions, Medical and Otherwise

**Published:** 1904-11

**Authors:** 


### SUPERSTITIONS, MEDICAL AND OTHERWISE.

C. C. Mapes, Louisville (*Medical Age*, July 25, 1904,), includes in his list a number of superstitions relating to mothers and infants:

"Mother's Friend" (an ointment) rubbed daily over the abdomen of a pregnant woman is believed to insure an easy and uncomplicated delivery.

As a pretty child is always desired, the mother is urged to look only at beautiful objects of art, etc., during her gestation. Many well-educated women have been known to invest in beautiful paintings and statuary and spend hours each day in contemplation thereof.

The prospective mother is advised to study higher mathematics and the sciences, that her child may possess extraordinary intelligence.

The position assumed at the beginning of labor, or during the latter part of the gestation, is believed to have an influence upon the child; if the mother extends her arms above her head, she is at once told to lower them, as such position will knot the funis around the baby's neck and produce its death.

Some women believe chloroform should not be taken to alleviate the pain of parturition, because it is flying in the face of Providence: "I will greatly multiply thy sorrow and thy conception; in sorrow thou shalt bring forth children."

That the child may possess a lovable disposition, the mother must under no circumstances get angry or quarrel during her pregnancy; indeed her mind must dwell only upon pleasant and agreeable occurrences.

If the child is a male the length of the penis at maturity is believed to be influenced by the length the funis is cut at birth.

The after-birth must be burned, in order to avoid the occurrence of after-pains.

To facilitate removal of the placenta, the patient is told to blow into a bottle, or to take snuff: or to hold a little salt in the left hand, double up the right hand into a funnel-shape and blow through it.

To prevent postpartum hemorrhage, the mother must take a "bite" of the placenta as soon as it has been delivered.

The obstetric superstition which has cost more lives than all the others combined is the fancied superiority of the old comforter as an absorbent of the liquor amnii, blood, urine, etc. The older, dirtier, and more often it has been used the more highly is it prized.

Instead of the baby being given mother's milk, immediately after it is born it is given a piece of fat bacon, a sugar teat, whisky and water, or some variety of tea.

The baby's fingernails are first to be bitten off, for if they are cut the child will surely be a thief. Do not cut the fingernails until they are one year old, or it will make the child steal.

The aperture through the compress applied to the funis must be burned through and not cut, otherwise the cord will suppurate.

The obstetrician is usually requested to examine the placenta and inform the mother whether or not she will bear further children.

The baby's eyes are washed with milk immediately after birth. This is a time-honored procedure and must under no pretext be omitted.

The husband of the pregnant woman is supposed to often suffer from "morning sickness," and is commiserated thereat.

Children are not infrequently weaned by the phases of the moon.

The Manx people believe that it will dwarf or wizen a baby if any one steps over it or walks around it.

In some parts of England people bind up the infant's right hand that it may have riches when grown.

In Yorkshire, England, a new-born babe is placed in a maiden's arms before being touched by any one else, in order to insure good luck.

In South America a book, a piece of money and a bottle of liquor are placed before the infant the day it is one year old, to ascertain its bent in life.

A baby is considered lucky in Scotland if it handles its spoon with its left hand, and it will be perfectly happy and successful if it has a number of falls before its first birthday.

In the north of England, when a child is taken from a house for the first time it is given an egg, some salt, and a small loaf of bread, and occasionally a piece of money to insure it against coming to want.

In Germany it is considered necessary that a child should "go up" before it goes down in the world, so it is carried up-stairs as soon as it is born. I case there is no up-stairs, the nurse mounts a table or chair with the infant.—*St. Louis Medical Review.*

